# Polysaccharide 3D Printing for Drug Delivery Applications

**DOI:** 10.3390/pharmaceutics14010145

**Published:** 2022-01-07

**Authors:** Alexandra Zamboulis, Georgia Michailidou, Ioanna Koumentakou, Dimitrios N. Bikiaris

**Affiliations:** Laboratory of Chemistry and Technology of Polymers and Dyes, Department of Chemistry, Aristotle University of Thessaloniki, GR-54124 Thessaloniki, Greece; michailidougeorgia18@gmail.com (G.M.); iwanna.koumentakou@gmail.com (I.K.)

**Keywords:** cellulose, chitosan, alginate, fused deposition modeling, extrusion-based printing, controlled drug release, personalized medicine, drug delivery, hydrogels

## Abstract

3D printing, or additive manufacturing, has gained considerable interest due to its versatility regarding design as well as in the large choice of materials. It is a powerful tool in the field of personalized pharmaceutical treatment, particularly crucial for pediatric and geriatric patients. Polysaccharides are abundant and inexpensive natural polymers, that are already widely used in the food industry and as excipients in pharmaceutical and cosmetic formulations. Due to their intrinsic properties, such as biocompatibility, biodegradability, non-immunogenicity, etc., polysaccharides are largely investigated as matrices for drug delivery. Although an increasing number of interesting reviews on additive manufacturing and drug delivery are being published, there is a gap concerning the printing of polysaccharides. In this article, we will review recent advances in the 3D printing of polysaccharides focused on drug delivery applications. Among the large family of polysaccharides, the present review will particularly focus on cellulose and cellulose derivatives, chitosan and sodium alginate, printed by fused deposition modeling and extrusion-based printing.

## 1. Introduction

Additive manufacturing, or, more commonly, 3D printing, is based on the layer-by-layer addition or deposition of a material in order to build a three-dimensional object. Not much noticed in the eighties, when they were first discovered, 3D printers have garnered a lot of attention in the 21st century due to their ease of use, versatility and wide array of applications [1,2]. In parallel to the fascination observed in the general public, the power of 3D printing has, not surprisingly, interested the academic and industrial communities. Indeed, 3D printing is a more flexible process, compared to conventional manufacturing techniques, and less wasteful [3]; it is even considered by some specialists as the next global industrial and manufacturing revolution [4,5]. The 3D printing industry is expected to reach a market size of $35 billion by 2024 [6]. A number of different 3D printing techniques and corresponding printers, appropriate for a wide range of materials (metals, plastics, ceramics, etc.), have now been developed, such as stereolithography, selective laser sintering (SLS), inkjet and extrusion-based 3D printing, fused deposition modeling (FDM), contributing to the success of additive manufacturing [3,7,8,9,10,11,12].

**Figure 1:**
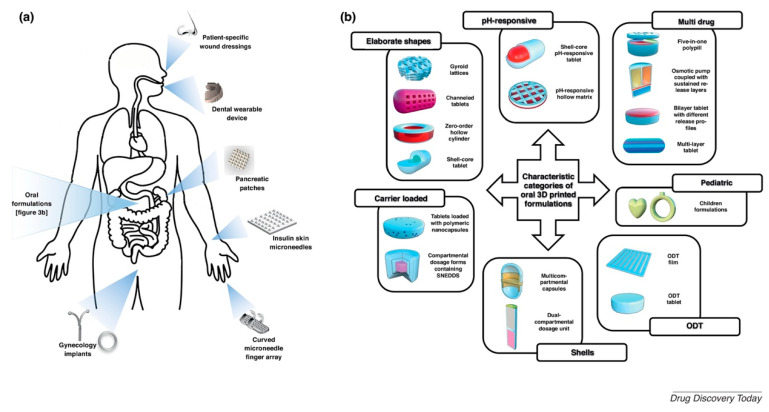
(**a**) Diversity of 3D-printed formulations and administration routes. (**b**) Variety of 3D-printed, orally administered solid dosage forms. Reprinted from [34], copyright 2018, with permission from Elsevier.

In the context of drug delivery, a wide variety of drug systems/formulations can be obtained by additive manufacturing, primarily diverse oral solid dosage forms, but also transdermal patches, vaginal and rectal delivery systems, as well as drug-loaded implants and surgical patches (Figure 1) [13]. For the time being, only one 3D-printed drug is commercially available, Spritam^®^, which is prepared by binderjet technology [14,15]. This limitation could be related to bottlenecks regarding the production speed, notably, which can be slower compared to conventional manufacturing techniques, but could also be due to regulation and health and safety issues [1,16]. In parallel to mass production, a real potential lies in the field of personalized medical treatment [17,18]. This applies, of course, to tissue engineering and regenerative medicine [19,20,21], where scaffolds with complicated structures can be printed according to the exact needs of the patient; but it is also a promising development for pharmaceutical treatments [22,23,24,25,26,27]. Currently, drugs benefit, to their highest efficiency, only a small percentage of patients [28]. This is related to the genetic material of each individual coupled with their lifestyle and environment. Personalized medicine aims at tailoring pharmaceutical treatment for each patient in order to increase the efficacy of their treatment. In this approach, 3D printing is a unique tool, allowing the preparation of personalized drug formulations [29,30,31]. In its simpler form, 3D-printing could allow drug-dose optimization (crucial for pediatric and geriatric patients), in other words, personalization of the amount of drug per tablet. Moreover, the geometry of the formulation could be modified, thus tailoring the release kinetics to a patient’s needs. Going a step further, two or more drugs could be combined in a single formulation with different release profiles to respond to multi-drug treatments, thus increasing the patient’s adherence to their treatment [16]. Some even envision the possibility of manufacturing medicines on demand at the point of care. For example, having common medications available in ink form at pharmacies or hospitals, which could then print personalized dosage forms. This is not feasible at the moment, due to the difficulty of guarantying the quality of the printed formulations, as well as regulatory questions, but, thanks to the progress of 3D printing, it is a conceivable development [15,32,33]. 

**Figure 2:**
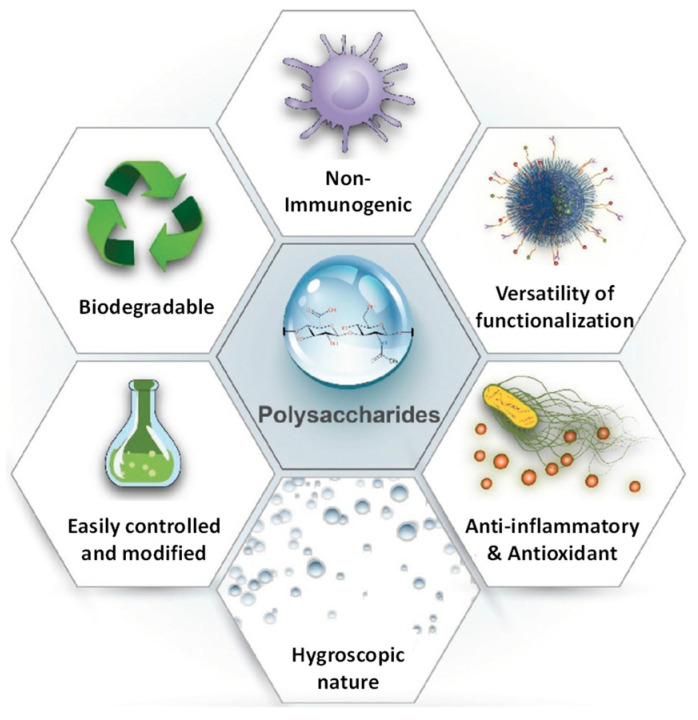
Schematic illustration of the unique physiochemical and biological properties of polysaccharides. Adapted from [38], Wiley, 2018.

Polysaccharides are natural polymers, derived from carbohydrates, that are quite abundant and easily accessible at a reasonable cost [35,36]. Carbohydrates are ubiquitously found in living organisms; they have structural functions and participate in several essential biological processes, such as energy production, storage and consumption. Additionally, they are involved in mechanisms of biorecognition. Polysaccharides are generally regarded as safe (GRAS) by the Food and Drug Administration (FDA) and are widely used in the food industry and as excipients in pharmaceutical and cosmetic formulations. Apart from their biocompatibility, polysaccharides have a low immunogenicity and are biodegradable, due to the susceptibility of the glycosidic bonds to enzymic hydrolysis. Moreover, they have abundant pending functional groups that can be modified to tailor their properties or used as anchoring points in the synthesis of polymer/drug conjugates. Due to these intrinsic properties (Figure 2), polysaccharides are widely investigated as matrices for drug delivery [23,37,38,39,40,41,42,43,44]. In this context, they can be formulated as nanoparticles, hydrogels, patches, lenses, filaments, etc. Finally, polysaccharides are often combined to other natural (e.g., proteins) or synthetic polymers to improve their properties, notably their lower mechanical properties.

Although an increasing number of interesting reviews on additive manufacturing and drug delivery are being published, there is a gap regarding the printing of polysaccharides. In this article, we will review recent advances in the 3D printing of polysaccharides focused on drug delivery applications, aiming at pointing out promising directions. This discussion will not be limited to the printing of drug delivery devices but will also enclose interesting reports concerning the development of printing inks. Among the large family of polysaccharides, the present review will focus on cellulose and its derivatives, chitosan (CS) and sodium alginate (Table 1) and on fused deposition modeling and extrusion-based printing. 

## 2. FDM and Extrusion-Based 3D Printing Techniques

The operating principle of 3D printing is the layer-by-layer fabrication of objects, using a digital design. Before printing, a 3D digital model of the object is created with a computer-aided design (CAD) software, offering the ability of designing various complex final structures. The structure is then “sliced” in 2D layers which are printed one by one, on top of each other, in order to finally afford the 3D object. Stereolithography, which is based on the selective photo-polymerization of a liquid resin, was the first technique developed for 3D printing technology [45]. Despite not often being used with polysaccharides, since these need to be modified in order to be susceptible to photo-polymerization, some very interesting reports have been recently published [46,47]. I Two other methods have mainly been employed for printing polysaccharides: fused deposition modeling (FDM), mostly for cellulose and its derivatives, owing to the good processing window between their glass transition temperature and the onset of thermal degradation temperature; and extrusion-based printing, which is appropriate for hydrogels. A short overview of the characteristics of these two printing techniques follows.

### 2.1. Fused Deposition Modeling (FDM)

**Figure 3:**
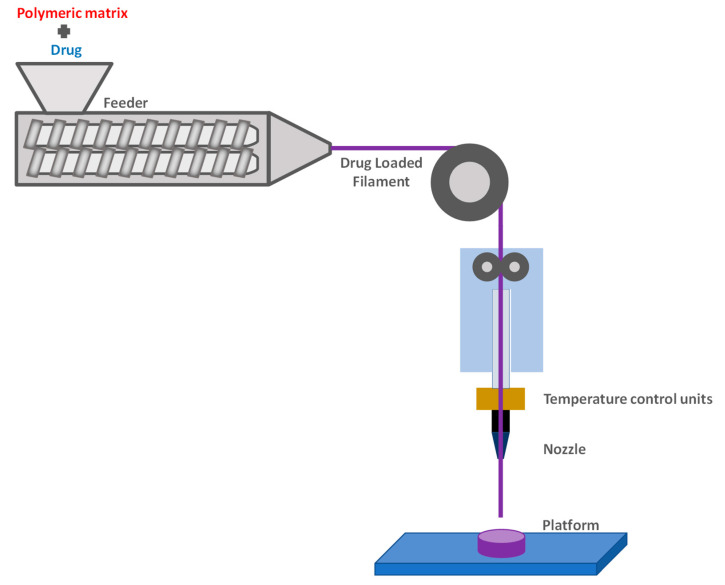
Simplified scheme illustrating the operating principle of hot-melt extrusion combined with fused deposition modeling.

Fused deposition modeling is a 3D printing technology that uses a continuous filament of a thermoplastic material for the fabrication of complete structures (Figure 3) [48,49,50,51]. The utmost advantage of FDM is the preparation of many shapes in a short time, with a low cost in comparison to traditional manufacturing processes [52]. FDM was developed by S. Scott Crump in the late 1980s and was commercialized in the early 1990s by (Stratasys Inc., Edina, MN, USA) [53]. Its setup is typically composed of a printhead freely movable in X and Y directions, a Z direction movable platform and a raw material in a cylindrical-shaped filament. The printhead consists of a heated nozzle where the polymer is softened/melted and laid down onto the platform as a layer according to the CAD design, where it cools down and hardens [54]. The following layers are deposited on top of the already printed layers and they then fuse with them, building the object in a bottom-up approach, while all the movements of the head and the raw materials are controlled by a computer.

There are various parameters affecting the quality of FDM 3D printed scaffolds namely the layer thickness, shell and infill pattern. Dimensional accuracy and surface roughness are among the most important characteristics to evaluate an FDM printed object [53]. Concerning dimensional accuracy, the glass transition temperature of the filament’s material determines the overall shrinking behavior of FDM components [55], while the immediate solidifying of the extruded material results in better dimensional accuracy. For the most part, filament materials applied in FDM printing are thermoplastics or, in general, materials able to melt when heated and immediately resolidify when cooling. PLA is commonly printed by FDM for pharmaceutical and medical applications but, progressively, other materials such as cellulose derivatives or Eudragit^®^ are being investigated [56,57,58].

Polymer filaments suitable for FDM are generally prepared by hot melt extrusion (HME). HME was first established in the early 1930s and was originally used for the manufacturing of plastics and rubber products in various shapes (films, fibers, granules) [59]. It is a very simple procedure: melted polymeric material is pumped through rotating screws via an extruder barrel resulting in a uniform filament. As expected, the properties of the extruded filament drastically affect the printing process. A fixed diameter throughout the length of the filament ensures uniform printing. Furthermore, the filaments must have an appropriate balance between flexibility and rigidity for continuous 3D printing. Indeed, soft filaments clog the FDM printer while brittle filaments break during printing [60].

FDM has a vast field of applications including automotive, aerospace, industrial and medical areas. For the printing of drug delivery systems, appropriate drug filaments are initially prepared by HME: the drug is dispersed in the polymeric matrix and uniformly distributed while extruded. Alternatively, filaments can be charged by immersion in a drug solution. The filament containing the drug is subsequently fed in an FDM printer to print the final drug-loaded object [55]. One of the assets of FDM is the lack of solvents, while a drawback is the temperature of FDM printing. Indeed, FDM takes place at high temperatures, since the polymeric material must be melted, resulting in a risk of degradation for the incorporated active pharmaceutical ingredients (API) [53]. Nevertheless, many research groups are studying the applicability of FDM in drug delivery systems for less heat-sensitive drugs, while research for the development of systems requiring lower temperatures is ongoing.

### 2.2. Extrusion-Based 3D Printing

**Figure 4:**
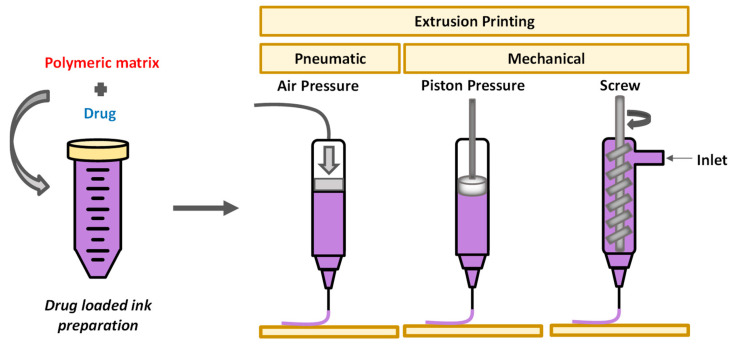
Extrusion-based printing systems.

In extrusion-based printing—also known as semi-solid extrusion printing, pressure-assisted microsyringe printing or direct ink writing—a semi-solid material, such as a paste or a gel, is extruded through a movable nozzle or needle under pressure and deposited on a platform (Figure 4) [61,62,63]. Similarly to FDM, after the completion of a single layer, the extrusion head either moves up or the build platform moves down for the deposition of the next layer. The materials to be printed, which are commonly called inks, along with the printing conditions, determine the performance of the final products [2].

The viscosity and the gel-forming behavior of materials are critically important in extrusion-based 3D printing [63,64]. In general, inks with shear-thinning behavior and fast recovery time are required [32]. To be more explicit, materials should be sufficiently viscous to retain their shape during the printing process but not too viscous, in order to obtain uniform strands and avoid nozzle clogging. After printing, the materials must undergo rapid gelation in order to avoid altering the printed construct [65,66]. Solidification can be obtained through the formation of non-covalent interactions and hydrogen bonds, crystallization, chain rearrangement, or by chemical crosslinking. The extrusion method enables compatibility with a large number of materials such as molten polymers, pastes, polymer solutions and hydrogels, the latter having received more attention as their viscosity can be carefully controlled [67].

Maintaining the structural integrity of the printed construct and achieving a high printing fidelity are key challenges in extrusion-based printing. Besides viscosity, other important parameters that should be optimized are: (1) the material output, (2) the needle diameter, (3) the nozzle feed rate, (4) the printer head speed, (5) the viscosity of extruded ink, (6) the temperature and (7) the distance between the nozzle and substrate. All these variables depend tightly on each other. For instance, if the needle diameter is decreased, the resultant strand diameter will decrease, and increased pressure might be needed to maintain a uniform strand deposition [68].

Extrusion-based 3D printing offers several advantages, including the ability to print a diversity of inks with a broad range of viscosities, due to the possibility to adjust the pressure used for extrusion as well as the possibility to co-print inks from different polymers, thus allowing easy access to spatially controlled multi-material scaffolds. Additionally, lower temperatures are used compared to FDM, rendering this printing technique particularly appropriate for the printing of drug- or cell-loaded scaffolds.

## 3. Cellulose and Cellulose Derivatives

Cellulose, the most abundant, naturally derived polysaccharide [69], can be produced from several renewable resources, mainly plants and bacteria. Bacterial cellulose (BC) is produced from the extracellular primary metabolite of various bacteria [70]. Plant-derived cellulose coexists with hemicellulose, lignin and extractives (nonstructural components with low molecular weight of lignocellulose), therefore, hydrolysis, chemical or enzymatic treatments are required for its isolation. The resulting cellulosic fibers are composed of microfibrils and each microfibril is composed of cellulose nanocrystals linked with cellulose nanofibers [71,72]. Due to its favorable properties (i.e., high crystallinity, elastic modulus, good mechanical properties, biodegradability and high water-holding capacity), cellulose is broadly utilized as a material in various pharmaceutical and biomedical applications [42,72,73,74].

**Scheme 1:**
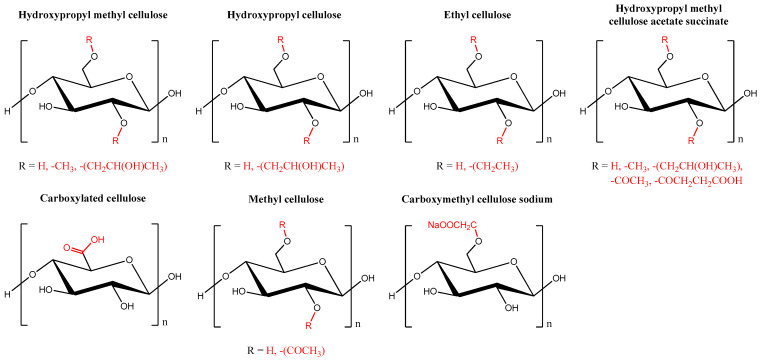
Structures of some common derivatives of cellulose.

Modification of polysaccharide structures is a common procedure for altering the initial properties of natural polymers. Chemical treatment of cellulose, mainly through esterification and etherification, yields polymeric derivatives with quite distinct properties [75], namely, improved solubility. Common cellulose derivatives are shown in Scheme 1 and include hydroxypropyl methyl cellulose (HPMC), hydroxypropyl cellulose (HPC), ethyl cellulose (EC), carboxymethyl cellulose (CMC) and methyl cellulose (MC) [76]. The solubility of cellulose ethers and esters depends significantly on the type of modification and on the degree of substitution [75]. For instance, highly substituted EC can be soluble in apolar organic solvents while HC and HPMC are amphiphilic. Some cellulose derivatives have thermoplastic properties (EC, HPC), while others exhibit thermoreversible gelation (MC, HPC). Most of these cellulose derivatives are commercially available in a range of molecular weights and substitution degrees. Due to the ease with which it can be derivatized along with its abundance, in nature, cellulose and its derivatives have been extensively utilized in biomedical and drug delivery systems. Depending on the specific properties of each derivative, both FDM and extrusion-based printing can be applied in the additive manufacturing of cellulose and its derivatives [76,77].

### 3.1. FDM Printing

Τhe aplicability of cellulosic derivatives in the FDM printing technique, along with the effect of the printed shape on drug delivery of various model drugs, has been evaluated by many groups. In general, FDM is applied to poorly water-soluble compounds and HME is used to uniformly disperse the drug into the cellulosic polymeric filaments [78]. As a result of the HME procedure, drugs are embedded in their amorphous phase into the polymeric filaments. As mentioned earlier, the utilized drugs must not degrade at elevated temperature during extrusion and printing processes.

The effect of the shell’s thickness and infill percentage on the release behavior of model drugs has been examined in many research papers. Zero order release tablets composed of hydroxypropyl methylcellulose (HPMC) containing acetaminophen were prepared by HME/FDM [79]. Tablets with a thick perimeter layer allowed for a controlled release while systems without an outer shell exhibited a fast release despite the dense infill pattern (100%), owing to the direct contact of the dissolution media with the tablet’s inner structure. Kadry et al. prepared diltiazem-loaded HPMC filaments and demonstrated that drug release percentage augmented as the infill percentage decreased [80]. When tablets with alternating drug-free and drug-loaded filaments were printed, a delayed release of diltiazem was achieved. Similar results were obtained with HPMC gastro-floating tablets containing dipyridamole (water-insoluble drug), printed by extrusion, with three different infilling percentages (30, 50, 70%.) [81]. All the formulations exhibited an 8 h gastro-floating sustained release. The in vitro release mechanism was mainly attributed to drug diffusion and polymer dissolution processes.

**Figure 5:**
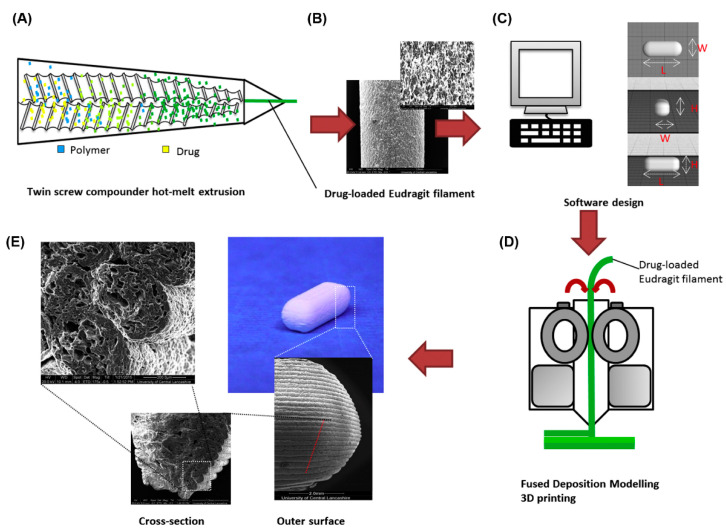
Fabrication of 3D-printed controlled-release theophylline tablet. (**A**) Schematic illustration of hot-melt extrusion to produce theophylline loaded filaments. (**B**) Drug-loaded filament. (**C**) Design of the capsule-shaped tablet. (**D**) Printing by fused deposition modelling. (**E**) 3D printed Eudragit tablets with a 200 μm layer thickness as shown in scanning electron microscopy (SEM) images. Reprinted from [85], copyright 2015, with permission from Elsevier.

Hydroxypropyl cellulose (HPC) has also been examined for the preparation of filaments, through HME, for FDM 3D printed drug delivery systems. Chai et al., prepared intragastric floating sustained release tablets with domperidone, a model drug that was loaded into the HPC filament [82]. The hollow shaped structures were printed, with a varying number of shells and infill percentages. Owing to the HME process, domperidone was entrapped in an amorphous phase into the cellulosic filament. In vitro and in vivo studies concerning the optimum tablet (2 shells and 0% infill percentage) indicated that the tablet’s floating ability was prolonged while sustained drug release was achieved. HPC has been also combined with vinylpyrrolidone vinyl acetate for the preparation of floating tablets loaded with cinnarizine [83]. HPC is a soft material, and its extrusion as a neat filament is difficult. The presence of vinylpyrrolidone vinyl acetate copolymer enhanced the mechanical strength of the drug-loaded material, allowing for successful printing. The pattern of the tablets had an impact on tablet floating behavior in the release medium as well as on the release profile of the model drug. Shell thickness and infill percentage affected the dissolution profile of cinnarizine: higher shell thickness and lower infill pattern resulted in higher cinnarizine release percentage and vice versa, offering an exceptional and accurate drug delivery system for personalized medicine. The printing behavior, through FDM, of HPC blends with other polymers commonly used in pharmaceutical formulations, such as Eudragit and polyethylene glycol (PEG), has also been assessed [84]. Theophylline, a thermally stable drug, was embedded in an amorphous state in the prepared filaments. During printing, a higher amount of HPC in the filament resulted in more brittle tablets. In vitro release studies showed a sustained release for over 10 h while, after the dissolution procedure, the tablets retained their original shape. In another study, the printing ability and the in vitro release behavior of neat HPC theophylline-loaded filaments were compared to three methacrylic polymers (Eudragit RL, RS and E) (Figure 5) [85]. The applied printing speed drastically affected the appearance of the tablets, while, during in vitro testing, the release profile of theophylline from 3D printed structures was more sustained, in comparison to release from non-printed drug-loaded filaments.

Ethyl cellulose (EC) is another cellulose derivative which finds application in drug delivery systems. Kempin et al. used EC and FDM for the preparation of 3D printed structures with a specified inner structure, and compared the 3D printed tablets obtained with various polymeric materials containing quinine as a model drug [86]. In vitro drug release studies from hollow cylinders demonstrated that drug release was heavily dependent on the polymeric matrix. Concerning specifically the EC tablet, 5% of the loaded drug was released over 100 days, making it an appropriate drug delivery system for very potent drugs, e.g., glucocorticoids, requiring prolonged administration.

Several cellulosic derivatives were further combined to enhance the in vitro drug release properties of the printed samples. Eleftheriadis et al., prepared mucoadhesive HPMC buccal films charged with ketoprofen [87]. In order to achieve unidirectional release, an EC-based backing layer containing lidocaine hydrochloride was incorporated. In vitro and ex vivo studies revealed that the inclusion of the backing layer did not affect the release behavior of lidocaine hydrochloride. Regarding ketoprofen’s dissolution profile, even though an extended release was attained, the incorporation of the backing layer significantly decreased the overall release percentage of the drug.

Extruded filaments designed for FDM printing should combine flexibility with good mechanical properties. In another study, filaments of varying EC/HPC ratios, containing carbamazepine and triethyl citrate as a plasticizer were prepared [78]. The optimum ratio between the two cellulosic derivatives was EC/HPC 2/1 and 20% of carbamazepine was released from the printed tablets over 24 h. Due to the low solubility of EC in aqueous media, the tablet’s shape was maintained throughout the release studies. EC and HPC blends were examined for the preparation of a core-shell pulsatile drug delivery system [88]. The printed shells were of various thicknesses (0.8, 1.2, 1.6, and 2.0 mm) and infill densities (50, 75, and 100%) while the core was composed of a compressed theophylline tablet. All the core-shell tablets revealed good floating behavior. The in vitro release behavior was mainly defined from shell thickness, while being also impacted by filament EC concentration.

Zhang et al., examined the optimum blends for the preparation of appropriate filaments from EC, HPC and HPMC with hydroxypropyl cellulose, PVP, Soluplas and Eudragit [89]. HPMC provides the required stiffness to the filament and consequently, the optimal filaments were composed of 45% HPMC and 20% of one of the other polymers (along with the drug (30%) and a disintegrator). Acetaminophen was uniformly dispersed into the extruded filaments while the printed tablets had a smooth surface and a strong, tight structure. In vitro release studies confirmed the enhanced and extended dissolution profile of the printed polymeric tablets.

The preparation of amorphous filaments composed of neat polymers or their blends, containing haloperidol through HME was studied by Solanki, et al. [90]. The examined polymers were Kollidon (poly(vinylpyrrolidone-vinyl acetate) copolymer), Kollicoat (polyvinyl alcohol-polyethylene glycol graft copolymer), Affinsiol (HPMC, HΜΕ 15) and hydroxypropyl methyl cellulose acetate succinate (HPMCAS). The drug-loaded filaments were 3D printed into tablets with different infill patterns (60 and 100%) through FDM, achieving rapid release of the model drug owing to their inner structure. The complete in vitro release was significantly faster from the tablets with a 60% infill pattern compared to tablets with a 100% infill pattern. In another study, the ability of HPMC and HPMCAS to form capsules, of various wall thickness (600 or 1200 μm), composed of separate compartments was assessed [91]. The capsule’s wall size greatly affects the release profile and delayed release was observed when capsules with 1200 μm wall thickness were tested in vitro. Moreover, the separate compartments are offering the ability to achieve in vitro release of multiple drugs with pulsatile release kinetics.

### 3.2. Extrusion-Based Printing and Other Printing Techniques

**Figure 6:**
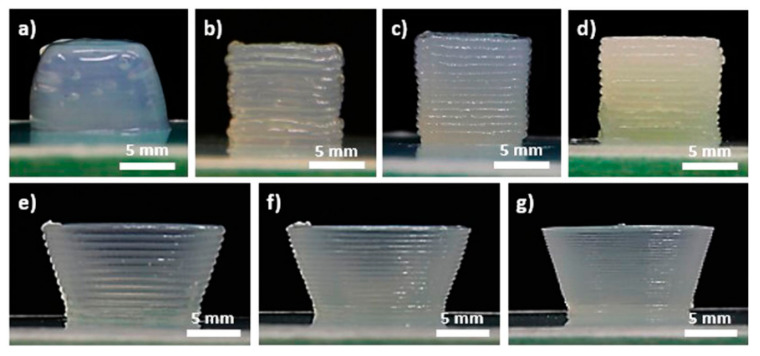
1 cm^3^ cubic cellulose nanocrystal (CNC) gel structures at (**a**) 11.8, (**b**) 15, (**c**) 20, and (**d**) 30 wt% are illustrated (nozzle tip size: 500 μm). Bowl structures printed with a 20 wt% CNC gel, from nozzle tips of (**e**) 500, (**f**) 400, and (**g**) 200 µm. Reprinted from [92], Sci. Rep. 2017.

Apart from FDM printing, cellulose and its derivatives have been subjected to additive manufacturing procedures through other 3D printing techniques. 3D printing of cellulose-based gels through pneumatic extrusion has been examined extensively. Li et al., prepared 3D printed cellulose nanocrystal aerogels with precize inner pore and architecture through direct ink write technique [92]. The concentration of cellulose affected the shape of the printed samples and higher definition was achieved by increasing the cellulose concentration in the gel. Moreover, employing a smaller nozzle tip during printing resulted in smoother lines and consequently more uniform surfaces (Figure 6). Gels composed of carboxymethylated and oxidized nanocellulose were successfully printed, forming dressings composed of 9 layers with an open porosity, able to suppress bacterial growth [93]. Poly(vinyl alcohol)/cellulose nanofibril hydrogels, formed due to the strong hydrogen-bond interactions between the two polymers, have also shown good printability [94]. The printed scaffolds were soft and flexible but retained their structural integrity, a combination of properties that is interesting for wound dressing materials. In this context, ascorbic acid release was studied with satisfactory results (80% release within 8 h).

Crosslinking of the samples is a solution regarding the preservation of their printed shape. For this reason, alginate (Alg) is often incorporated to cellulose inks. Indeed, Alg is easily crosslinked in the presence of calcium ions and can thus contribute to the structural integrity of printed objects. Olmos-Juste et al. prepared Alg/cellulose nanofibers porous scaffolds loaded with curcumin [95,96]. A content of cellulose nanofibers as low as 3–5% was able to maintain the structure and shape fidelity along with printing accuracy of the scaffolds providing them with enhanced mechanical strength. The concentration of the nanofibers was crucial for the in vitro release of curcumin since higher concentration resulted in smaller porosity of the scaffolds and consequently to a more sustained release profile. Likewise, sustained release was obtained when clindamycin was released from nanofibril cellulose, Alg and CMC 3D printed coatings deposited onto titanium alloy and stainless-steel substrates [97]. The printing of TEMPO-oxidized cellulose nanofibrils was studied for the preparation of aerogels for wound dressing applications [98]. Alginate was incorporated into the oxidized cellulose gels to allow ionic crosslinking with calcium chloride (CaCl_2_); results demonstrated that the higher the concentration of the crosslinker applied, the higher the definition of the printed samples. Instead of alginate, Cernencu et al. took advantage of the crosslinking properties of pectin (Pec) and studied the preparation of blends composed of pectin and TEMPO-oxidized cellulose nanofibrils [99]. The presence of Pec improved the printability of the inks, while the presence of oxidized cellulose conferred enhanced rehydration properties to the printed samples. Wei et al. showed that adding laponite nanoclays to a TEMPO-oxidized BC/sodium alginate printing ink allowed for enhanced structural stability, improving the compressive strength as well as the hydrolysis behavior of the printed hydrogels, while delaying the release of bovine serum albumin (BSA) [100]. Overall, a long-term in vitro release of the protein was achieved.

**Figure 7:**
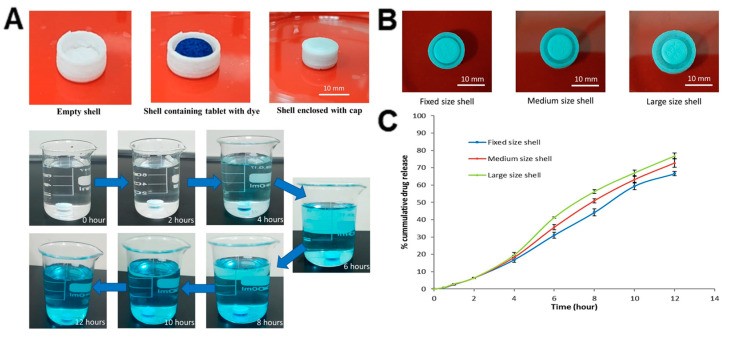
(**A**) Encapsulation of the propranolol HCl tablet soaked with color dye (methylene blue) inside the 3D printed controlled-release shell, and gradual increase of the intensity of the color solution showing visually the release of methylene blue from the 3D printed shell. (**B**) Different sizes of 3D printed controlled-release shells illustrating the gap/space between the enclosed tablet and the shell wall. (**C**) Cumulative release of propranolol HCl with phosphate buffer (pH 6.8) as dissolution media at temperature 37 ± 0.5 °C from 3D printed controlled-release shells of different sizes. Reprinted from [102], Polymers 2020.

Analogous results emerged from the work of Ahlfeld et al., where an ink composed of alginate, methylcellulose and laponite was prepared [101]. The addition of the nanoclay resulted, on the one hand, in easy and smooth extrusion of the ink along with high printing precision and shape retention of the samples and, on the other hand, contributed to a more controlled and sustained release of BSA and vascular endothelial growth factor (VEGF) loaded into the ink. In another study, a cellulose acetate porous polymeric shell was printed, with an extrusion-based 3D printer, for a propranolol HCl immediate-release tablet (Figure 7) [102]. According to the authors, increasing the size of the shell resulted in higher dissolution rates, attributed to the higher amount of dissolution medium that can penetrate in the interior of the tablet, and that dissolves larger amounts of the drug.

Huan et al., prepared gels composed of cellulose nanofibrils, alginate and PLA designed for 3D printing [103]. Different infill patterns were investigated. The prepared gels exhibited shear thinning behavior while cellulose nanofibrils acted as stabilizers, ensuring the printability of the gels. The presence of randomly distributed PLA was responsible for the maintenance of the structure. Liu et al. prepared implantable cancer tissue patches, composed of gelatin methacryloyl and carboxymethyl cellulose sodium [104]. The presence of CMC increased the viscosity of the final ink which was 3D printed in three different shapes (cylinder, torus, gridlines). PEGylated liposomal doxorubicin was incorporated in the hydrogel and photo-polymerized. In vitro release studies demonstrated that the torus shaped hydrogels had the larger surface area, leading to enhanced in vitro release of PEGylated liposomal doxorubicin. The same technique was applied for the development of high levetiracetam-loaded tablets, composed of HPC [105]. The tablets were printed in three different shapes (cylinder, torus and oval) in various concentrations. The most promising extrudability was attained with paste concentration varying between 0.30–0.50 g/mL. In vitro release studies revealed that the printing structure is heavily affecting the dissolution profile of the tablets. The highest in vitro release percentage was measured in the torus-shaped tablet with 50% inner infill percentage (97.45% the first 2 min). Individualized orodispersible HPMC films loaded with varying amounts of levocetirizine hydrochloride were also prepared by semi-solid extrusion [106]. Films printed with 50% infill pattern displayed a faster dissolution profile in comparison to compact films (100% infill), whereas lower dose films were able to reach in vitro release up to 80% in 1 min.

The liquid deposition modeling (LDM) extrusion process is based on the direct deposition of a homogeneous dispersion using a highly volatile solvent, ensuring its fast evaporation during wet filament deposition and rapid formation of rigid 3D microstructures [107]. Mohan et al., printed laminated films composed of calcium carbonate and nanocellulose, containing 5-fluorouracil (5-FU), through LDM [108]. In vitro release studies in a simulated colon environment indicated that the initial burst release of 5-FU was restrained, and a time-controlled release was observed. CaCO_3_ porous structure resulted in high surface area whereas, nanocellulose was responsible for the controlled release of the drug. Selective laser sintering (SLS) is a relatively new rapid prototyping process that allows the generation of precise complex 3D structures by solidifying successive layers of powder materials on top of each other [109]. In their work, Fina et al., examined the in vitro release behavior of paracetamol from 3D printed structures of EC, through SLS technology [110]. The in vitro release was assessed in stimulated gastrointestinal tract conditions. The gyroid lattice design enhanced release, although a complete dissolution of the drug was not achieved, due to the insoluble nature of EC.

**Figure 8:**
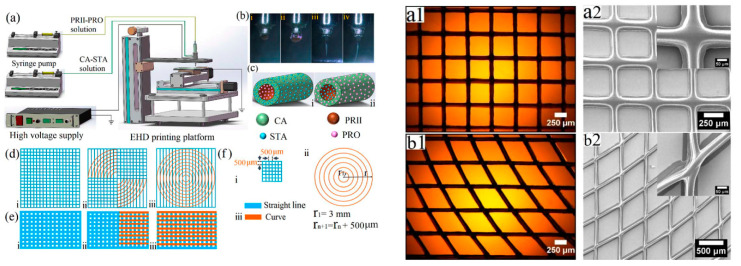
Left: (**a**) Schematic diagram of core-shell electrohydrodynamic 3D printing system; (**b**) Forming process of continuous core-shell stable-jet mode at different voltages: (i) 0 kV, (ii) 1 kV, (iii) 2.5 kV (front view), and (iv) 2.5 kV (side view), (printing: 20 mm/s); (**c**) schematic illustration of the core-shell fibers, CA (cellulose acetate), PRII (polyacrylic resin), STA (stachyose), PRO (proteoglycan); (**d**) top view of patterned fibrous membrane with various geometries—(i) square, (ii) semi-circle, (iii) whole-circle; (**e**) front view of the patterned membrane corresponding to i, ii, and iii of (**d**); (**f**) membrane characteristics—(i) space between two printed fibers; (ii) radius of curves, and (iii) printed fiber with straight and/or curved line. Right: Indicative optical images (**a1**,**b1**) and scanning electron images (**a2**,**b2**) of printed fibers loaded with STA and PRO with various patterns—(**a1**,**a2**), square; (**b1**,**b2**), semi-circle. Reprinted from [111], Biofabrication 2020.

Electrohydrodynamic 3D printing is applied for the development of complex structures with accurately stacked and aligned fibers, using concentrated organic/polymer solutions. Through this technique, core shell structured fibrous membranes, loaded with stachyose and proteoglycan, were prepared by coaxial printing (Figure 8) [111]. Stachyose is a tetrasaccharide able to improve the proliferation of probiotics whereas proteoglycan is a protein that inhibits the growth of pathogenic bacteria on the human gastrointestinal tract. The core was composed of fibered cellulose acetate where stachyose was encapsulated, while proteoglycan was incorporated in the shell that consisted of a polyacrylic resin. Fibers with various geometries (square, semi-circle, and whole-circle membrane) were printed and their well-aligned structure was evidenced by scanning electron microscopy (SEM) (Figure 8). In vitro release in simulated intestinal fluid (pH = 7.4) showed a biphasic drug release profile with a burst release during the first 12 h and a slower sustained release up to 3 days. After 3 days, the release of all membranes was nearly 95%.

Inkjet printers employ heat or mechanical compression to eject ink drops. Thermal inkjet (TIJ) 2D printers are exploiting small air bubbles, generated while heating the printhead, which collapse and provide pressure pulses to eject the ink drops [112]. Oro-dispersible films composed of glycerol and HPMC, for the delivery of hypothyroidism triiodothyronine (T3) and thyroxine (T4) were obtained by TIJ printing [113]. The drugs were dissolved in a solvent mixture and printed on the surface of the glycerol/HPMC substrates. This strategy enables the preparation of multi-drug formulations with personalized therapeutic dosages since dose modification is achieved by altering the length of the printed shapes during printing.

## 4. Chitosan and Chitosan Derivatives

**Scheme 2:**
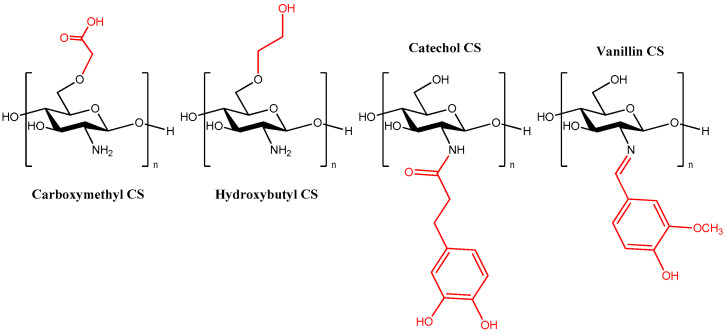
Chitosan derivatives that have been used for 3D printing.

Chitosan (CS) is a versatile polysaccharide, bearing structural similarities to glycosaminoglycans, with numerous applications in many research fields including pharmaceutical and biomedical fields [114,115,116,117]. Apart from the properties mentioned in the introduction, that are common to most polysaccharides, CS exhibits antimicrobial and mucoadhesive properties, which are attributed to the positive charge of the amino groups [117,118]. Several CS-containing products have already been approved by the FDA and 3D printing of chitosan, chitosan derivatives (some examples discussed in this article are illustrated in Scheme 2), and chitosan blends has already attracted interest [119,120,121,122,123]. Pure CS hydrogels have been printed, but an additional crosslinking step is usually required to further stabilize the printed constructs. To improve the characteristic of the CS printed hydrogels, CS has been blended with natural polymers, other polysaccharides, such as sodium alginate (Alg) or pectin (Pec), and proteins, e.g., gelatin (Gel), as well as synthetic polymers, notably poly(lactic acid) (PLA) and polycaprolactone (PCL).

### 4.1. Extrusion-Based Printing of Chitosan Inks

**Figure 9:**
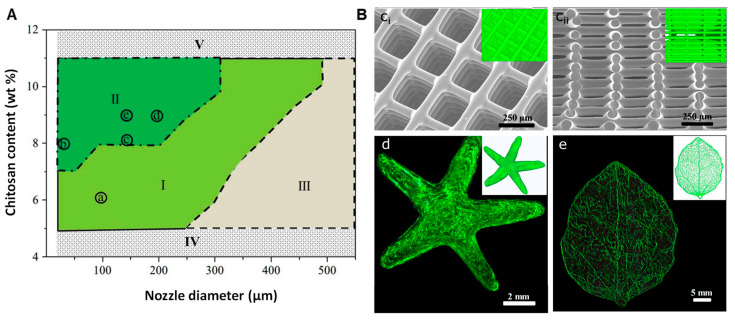
(**A**) Process map illustrating the ranges of chitosan contents and nozzle diameters compatible for the fabrication of various types of microstructures I, zone for 1D filament; II, zone for 2D filament array or 3D structure; III, zone where chitosan solutions lose shape fidelity; IV, zone where chitosan solutions are too diluted for the process; V; zone where the chitosan solutions are too viscous to be printed. The letters c–e represent the fabrication parameters of different structures shown on the right. (**B**): c_i_ and c_ii_ SEM top and side views of a 3D chitosan scaffold with square pore size; d and e, fluorescent microscopy images of a 3D printed starfish and a 3D printed leaf; respectively. Inset images show the corresponding CAD models. Adapted with permission from [124]. Copyright (2018) American Chemical Society.

Wu et al. have studied pure chitosan inks and the conditions to obtain 1D, 2D and 3D hydrogel constructs [124]. Interestingly, the authors constructed a map (Figure 9) illustrating the type of constructs that could be obtained depending on CS concentration and the printing nozzle’s diameter, along with the conditions where printing was not successful. Sadeghianmaryan et al. demonstrated that swelling and degradation were more important in air-dried CS scaffolds compared to warm and vacuum dried scaffolds [125]. The incorporation of citrate-modified iron nanoparticles in a printed CS scaffold was investigated by Lin et al. [126]. Covalent bonds were formed between the citrate groups of the nanoparticles and the CS chains, immobilizing the nanoparticles in the hydrogel. The encapsulation and release of bovine serum albumin (BSA) was further studied by the authors.

**Figure 10:**
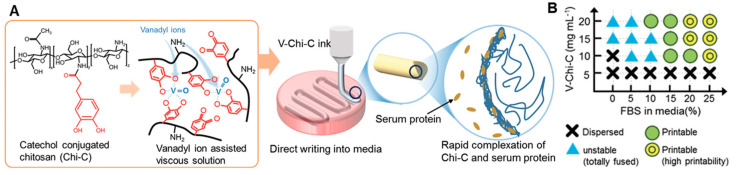
Catechol-chitosan ink with vanadyl ions (V-Chi-C). (**A**) Preparation of the ink and 3D printing process. (**B**) The printable window using the combination of V-Chi-C solution prepared as a function of concentration and the culture media containing fetal bovine serum (5, 10, 15, 20 or 25%). Reproduced from [129] with permission from The Royal Society of Chemistry.

Marques et al. developed an ink composed of biphasic calcium phosphate powders (45%), CS and genipin, to print levofloxacin-delivering scaffolds for bone regeneration [127]. A double crosslinking was applied—ionic due to calcium ions and covalent with genipin. Levofloxacin-loaded scaffolds showed an early and fast drug release and effectively inhibited bacterial growth in in vitro experiments. Liu et al. fabricated 3D scaffolds from CS and CS crosslinked with pectin and genipin and studied the drug release of pentoxifyllin, a drug used clinically to reduce inflammation [128]. The chitosan solutions were directly printed in a NaOH solution and pentoxifyllin was adsorbed on the scaffolds in a separate step. Compared to CS freeze-dried scaffolds, fully interconnected and well controlled pores were obtained by 3D printing. Pentoxifyllin release was fast with a ca. 85% release within 6 h. Lee et al. reported a catechol-modified CS ink that was directly printed in fetal bovine serum (FBS)-containing media (Figure 10) [129]. Stable complexes that did not dissolve were formed due to the interactions developed with proteins. In the presence of vanadyl ions, metal coordination bonds were additionally formed between vanadium and catechol, acting as crosslinks and further strengthening the scaffold. Though not studied yet, this ink showed promising characteristics for drug delivery or tissue engineering applications.

**Figure 11:**
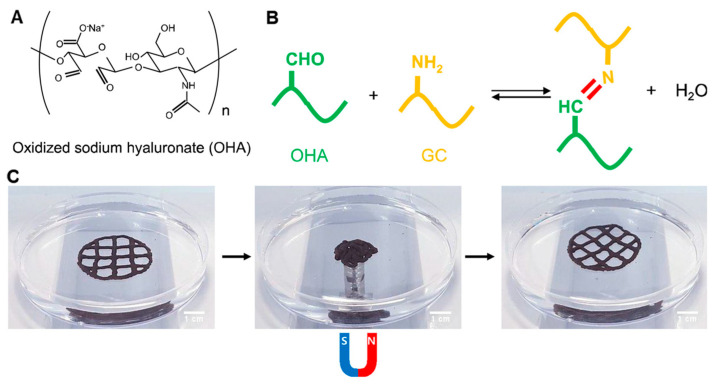
(**A**) Chemical structure of oxidized sodium hyaluronate (OHA). (**B**) Schiff base formation between OHA and glycol chitosan (GC). (**C**) Change in the 3D-printed structure by application of a magnetic field. The original shape was recovered after removal of the magnetic field. Reprinted with permission from [130], Copyright 2020, Elsevier.

Oxidizing polysaccharides affords polymers bearing aldehyde moieties. These groups can easily react with the amino groups of chitosan, forming reversible imine bonds (Schiff bases), resulting in the formation of hydrogels. Ko et al. reported a self-healing ferrogel (magnetic field-sensitive hydrogel containing iron oxide nanoparticles (ION)) prepared from glycol CS and oxidized hyaluronic acid (HAc), which was used as an ink for 3D printing [130]. By applying a magnetic field, the 3D printed construct could lose its shape while the original shape was recovered when the magnetic field was removed, as illustrated in Figure 11. Though not yet studied, this polysaccharide-based ferrogel demonstrates an excellent potential for further drug delivery applications. Michailidou et al. examined the formation of a Schiff base derivative of CS with vanillin (VACS) and the further formation of miscible blends of the VACS derivative with neat CS [131]. Interactions occurring between the hydroxyl groups of vanillin of VACS and the free amino groups of CS lead to viscous inks with shear thinning behavior and increased synergic interaction, appropriate for 3D printing applications.

CS is often combined with sodium alginate (Alg), an anionic polysaccharide, resulting in the formation of polyelectrolyte complexes due to the ionic interactions that evolve between the amino groups of CS and the carboxylic acid groups of Alg at low pH. Liu et al. reported an original approach were CS and Alg were initially mixed at neutral pH [132]. As a result of CS swelling, the solution exhibited a viscosity appropriate for printing. After printing, aqueous HCl was sprayed on the printed construct, the pH was lowered and polyion complexes with Alg were formed due to the protonation of CS amino groups, stabilizing the printed structure and maintaining the structural integrity without any supplementary crosslinking step. Depending on the targeted application, the properties of the final constructs could be tuned by the CS/Alg ratio.

Pectin (Pec) is another anionic polysaccharide (Table 1), extensively used in the food industry as a thickening agent. Similarly to sodium alginate, due to its carboxylic groups, pectin forms polyelectrolyte complexes with CS. Michailidou et al. assessed the printability of CS/Pec hydrogels [133]. The addition of Pec increased significantly the viscosity of the CS/Pec hydrogels and consequently improved the shape fidelity of the printed constructs. The ink composed of 5% CS and 10% Pec exhibited the best printability. It was evidenced by IR spectroscopy that the ionic interactions between the two polysaccharides were assisted by the formation of H bonds. The printed samples could maintain their structural integrity without further post-printing processes. Three-dimensionally printed lidocaine hydrochloride-loaded scaffolds for wound dressing based on a CS/Pec thermosensitive hydrogel were reported by Long et al. [134]. According to the authors, gelation is based upon the formation of H-bonds rather than ionic interactions, because at the pH used Pec chains are not charged. Upon heating, the H-bonds are cleaved, imparting a temperature-controlled gelation. A 1 h burst release of lidocaine was observed, followed by a sustained release for the following 4 h, with the cumulative release reaching approximately 90% at the end of the experiment. This release behavior is interesting when aiming to provide an effective and rapid pain relief in cases of fresh wounds. Hyaluronic acid, another linear anionic polysaccharide (Table 1), can also form a polyelectrolyte complex with chitosan, similarly to pectin. Maiz-Fernández et al. have reported self-healing CS/HAc hydrogels based on the formation of polyelectrolyte complexes and optimized their concentrations to obtain printable hydrogels [135]. Sodium diclofenac and rifampicin were further charged in the gels by absorption. It was observed that rifampicin release was slower compared to sodium diclofenac due to the large size of the molecule and its lower solubility.

Carboxymethyl chitosan/starch blends have also been studied for the 3D printing of wound dressing patches [136]. Different ratios of the two polysaccharides were investigated and it was demonstrated that as the starch ratio increased a more sustained release of mupirocin, a topical anti-infective, was observed, due to the lower hydrophilicity of the patch. Nevertheless, when the efficacy of those patches was tested against *S. aureus*, the patches with higher CS concentration showed a larger zone of inhibition at all studied time points.

As polysaccharides, proteins are natural polymers, often charged, which can interact ionically or by H-bonds with chitosan. Three-dimensionally printed scaffolds that have been prepared by CS/protein or protein-like polymer hydrogels, such as poly(gamma-glutamic acid) will be further discussed.

Gelatin (Gel) is an anionic polymer, produced by the partial degradation of collagen. When combined, Gel and CS form hydrogels with thermoresponsive properties. The chains of the two polymers form polyelctrolyte complexes or interpenetrated networks, resulting in gelation. These hydrogels are usually printed below the temperature of gel to liquid transition and gelation is triggered, after printing, either by lowering the temperature (i.e., the scaffold is printed on a cooling platform) or via the addition of crosslinkers. Roehm et al. evaluated the printability of a CS/Gel thermosensitive hydrogel, formed in the presence of β-glycerophosphate [137]. Increasing CS concentration resulted in shorter gelation time and lower gelation temperature. CS/Gel hydrogels, crosslinked by tripolyphosphate (TPP) were studied by Fischetti et al. [138]. TPP was selected instead of genipin or glutaraldehyde, owing to its lower cost and non-toxicity, respectively. In terms of stability and compatibility, the hydrogel prepared with a 1/2 CS/Gel ratio (2% and 4% *w*/*v* respectively) was optimal. Chen et al. developed CS/Gel/HAp composite pastes for 3D printing [139,140]. After printing, the scaffolds were coated with multiple layers of CS and sodium hyaluronate. Bone morphogenic protein (BMP) 2 and vascular endothelial growth factors (VEGF) were simply loaded by adsorption [139]. The obtained scaffold had an important porosity and demonstrated a sustained release of both BMP-2 and VEGF over 14 days.

**Figure 12:**
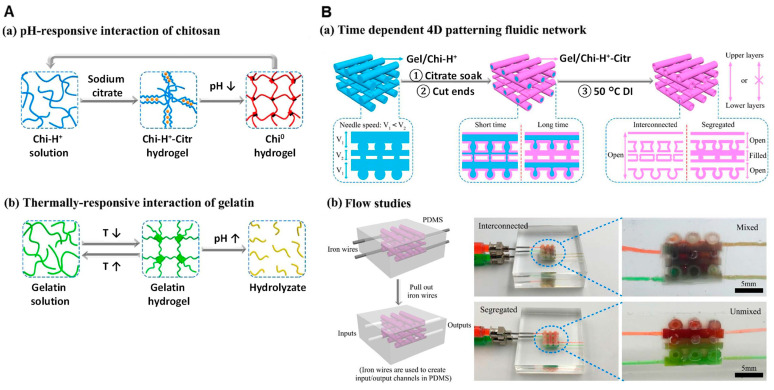
(**A**) Stimuli-responsive crosslinking of (**a**) chitosan and (**b**) gelatin (**B**) 4D patterning can determine whether a fluidic network is interconnected or segregated. (**a**) Schematic of the 4D patterning process to generate an interconnected or segregated fluidic network. (**b**) Functional evidence showing flow of the red and green input dyes is either mixed or segregated in an interconnected or segregated fluidic network. Here 4D patterned structures were embedded into PDMS in order to fix the syringe needles to avoid leakage of liquid during perfusion and clearly show the colors of liquids in each input and output. Reprinted with permission from [141], IOP Publishing.

Although not applied to the delivery of API yet, Wen et al. have reported an elegant hierarchical patterning approach for the elaboration of complex multidimensional structures based on a stimuli-responsive hydrogel composed of a CS/Gel interpenetrated network (composition: 7% CS and 18% Gel) (Figure 12A) [141]. When the hydrogel was exposed to sodium citrate it was rendered insoluble in warm water due to CS crosslinking, in contrast to the initial hydrogel. To obtain 2D structures, a sodium citrate solution was printed on a CS/Gel printed film. The hydrogel in contact with sodium citrate was selectively crosslinked and the non-crosslinked hydrogel was removed with warm water affording a precisely defined construct consisting of the crosslinked CS/Gel hydrogel. Three-dimensional constructs were prepared with a layer-by-layer approach while tubular structures were obtained by immersing 3D CS/Gel constructs in a sodium citrate solution. By controlling the duration of immersion, sodium citrate diffusion in the hydrogel was controlled and an additional tuning parameter was introduced (Figure 12B).

Collagen (Col) is a structural protein composed mainly of glycine, proline and hydroxyproline residues. Cheng et al. reported a BMP delivery system based on BMP-loaded graphene oxide nanoparticles, which were further incorporated in a Col/CS hydrogel (10/1 *w*/*w* ratio) [142]. It was demonstrated that BMP-7 was successfully released from the printed structure, in useful concentrations, over at least 30 days, without burst release and in a rather regular fashion.

Pisani et al. reported the preliminary investigation of a bio-ink composed of CS and poly(gamma-glutamic acid) (PgGA) [143]. As mentioned above, CS and PgGA form polyelectrolyte complexes. In the printed scaffolds, SEM images displayed porous interconnected structures, with porosity depending on CS concentration. Silk fibroin is an interesting fiber, that has attracted attention in a range of applications, especially when good mechanical properties are required [144,145,146]. It was found that silk fibroin nanofibers enhanced the shear-thinning behavior of CS solutions and improved their printability (higher printing fidelity) [147].

Poly(vinyl alcohol) (PVA) and poly(ethylene glycol) (PEG) are two water-soluble synthetic polymers frequently used for pharmaceutical and biomedical applications [148]. PVA/CS filaments were prepared by HME for the 3D printing of films by FDM, for the oromucosal drug delivery of diclofenac sodium (model hydrophilic drug) [149]. It was shown that CS improved the mucoadhesion and drug permeation properties of the films. In the ex vivo studies that were performed, it is noteworthy that the amount of diclofenac sodium extracted from buccal tissues when CS was present in the film was significantly higher than in its absence. This was attributed to the interactions of cationic CS with the buccal membrane which resulted in the reorganization of the tight protein junctions and improved permeation. In these films, the content of CS was rather low, however, a printable CS/PVA/HAp hydrogel with a 2.5/1 CS/PVA mass ratio and various amounts of hydroxyapatite has been reported [150]. The hydrogel exhibited good mechanical properties due to the intermolecular interactions between the two polymers. After printing, the structure was crosslinked with sodium hydroxide and a 36-layer scaffold was successfully fabricated. It was observed that printing quality improved with increasing the hydroxyapatite content. Finally, the best scaffold was further loaded with BMP-2.

Fabrication of personalized dressings for wound treatment is one of the principal applications of 3D printing. In this day and age, wound dressings do not simply protect or cover a wound but participate actively in the healing process. Indeed, the porous structure of dressings and the sustained release of anti-inflammatory or other drugs could facilitate healthy and optimal tissue regeneration. Hafezi et al. studied a CS/PEG hydrogel, crosslinked with genipin, which is less toxic than glutaraldehyde, for printing applications [151,152]. In this work, the CS/PEG ratio was used to control crosslinking, and thus viscosity. The optimal hydrogel was obtained for a 1.2% CS concentration, at a 1:1 CS: PEG ratio and crosslinked with 1% wt genipin [152]. Multilayered films, loaded with fluorescein sodium as a model water-soluble compound, were further prepared by 3D printing for chronic wound healing [151]. PEG was shown to improve the flexibility of the obtained films. Due to the high solubility of fluorescein sodium, a very fast release was observed (67% within the first hour), but a better control over release is expected for less soluble active ingredients (ideally, a release over 24 to 48 h is more helpful for patients as it avoids the need to change the dressing frequently).

### 4.2. Incorporation of Chitosan Micro/Nanoparticles in Printing Inks

CS nanoparticles have been extensively examined as vehicles for sustained drug delivery. Therefore, it is not surprising that drug-loaded CS micro- and nanoparticles were incorporated in 3D printing inks to achieve a more sustained, better controlled, targeted release [153]. An interesting case of study is the incorporation of human bone morphogenic proteins (rhBMP) in nanoparticles. Printed scaffolds for tissue engineering are often loaded with factors that will assist/stimulate tissue reconstruction, and, in the context of bone regeneration, rhBMPs are among the most popular osteoinductive agents. Deng et al. elaborated a PLGA/nanohydroxyapatite (nanoHAp)/CS scaffold where bone regeneration was improved due to the sustained and controlled delivery of rhBMP-2 [154]. rhBMP-2 was loaded into CS microspheres, which were further encapsulated in a temperature-sensitive CS hydrogel obtained from CS and β-glycerophosphate. PLGA/nanoHAp and the CS hydrogel loaded with rhBMP-2 microspheres were combined via a two-barrel 3D bioprinter, with one barrel for PLGA/nanoHAp functioning at 130 °C and the other one for CS hydrogel at 4 °C. In the release studies that were performed, a sustained release over 30 days was achieved. These good results were attributed to the “double” encapsulation of rhBMP-2, in the microspheres initially and in the hydrogel afterwards. Wang et al. prepared CS microspheres containing rhBMP-2 which, with the aid of a collagen solution, were coated on 3D printed porous HAp scaffolds [155]. A sustained release over 21 days was observed and, at all time-points, the rhBMP-2 concentration in the release medium was higher than the minimum required BMP-2 amount to achieve a biological result. Along the same line, BSA or VEGF were encapsulated in CS/dextran microparticles, and, after freeze-drying, incorporated in a tricalcium phosphate paste, that was further plotted [156]. A low cumulative drug release was observed, over 2 days for BSA and 14 days for VEGF, which was attributed to the low porosity of the scaffolds. Nevertheless, VEGF bioactivity was maintained, and despite its low concentration, VEGF had a positive effect on cell growth in in vitro experiments.

Chitosan and β-cyclodextrin/propolis extract inclusion complexes were loaded in pectin-based inks, containing Manuka honey, to develop wound dressings by 3D printing [157]. Due to the opposite charges of CS and Pec, topical crosslinking of Pec was observed around the inclusion complexes, affecting the degradation and mechanical properties of the pectin printed films. The incorporation of the propolis inclusion complexes enhanced the antibacterial and wound-healing properties of the dressings. Vadodaria et al. reported the elaboration of surfactant-polyelectrolyte complexes using a valvejet 3D printer [158]. These complexes were obtained by printing alternating layers of sodium dodecyl sulfate (surfactant) and CS (polyelectrolyte). The obtained construct had a good structural integrity and homogeneous distribution of surfactant-polyelectrolyte microcapsules (diameter around 300 μm) throughout its structure. According to the authors, these microcapsules could be used for the delivery of APIs.

### 4.3. Coating of Printed Constructs with Chitosan

CS coating is an accessible strategy to enhance the adhesion, biocompatibility or biodegradability of particles or scaffolds and to increase the control over the sustained delivery of pharmaceutical ingredients. Vordran et al. presented a multijet 3D printing system which allowed low temperature processing and good accuracy over the spatial localization of bioactive ingredients in the 3D printed scaffolds [159]. The four inkjet printheads of the multijet system contained solutions of phosphoric acid (binder), bioactive ingredients (vancomycin, rhBMP-2 and heparin), chitosan hydrochloride (1% wt) and TPP (crosslinker), respectively. These solutions were simultaneously printed on tricalcium phosphate powder (drop-on-solid printing). As a result, a bruschite (CaHPO_4_)-based drug-loaded core was obtained, with a CS external layer. The successful preparation of this system is, among others, due to the fast gelation of CS induced by its reaction with the TPP polyanions. The CS coating layer acted as a polymeric barrier that contributed to control the diffusion and thus the release of the bioactive substances.

A PLA 3D printed tablet with a Eudragit^®^ (a pH-responsive polymethacrylate polymer) bottom layer was designed for the sustained and targeted delivery of 5-fluorouracil (5-FU) in acidic environments for the treatment of colorectal cancer [160]. 5-FU was encapsulated in CS-coated Alg beads which were loaded in the PLA/Eudragit 3D printed tablet. CS was used due to its mucoadhesive and pH-sensitive properties, in order to improve the controlled delivery of 5-FU at a pH value corresponding to the colonic environment. Similarly, a CS layer was coated on a 3D printed Alg scaffold loaded with diclofenac, an anti-inflammatory drug, aiming to retain more drug within the scaffold and slow down its diffusion during release [161]. An amount three times larger of diclofenac was loaded in the coated sample, demonstrating that the CS barrier could indeed prevent drug loss during scaffold preparation. Moreover, the CS layer delayed the onset of drug release, while an overall slower release pattern was also observed, due to the crosslinked polymeric network formed between CS and Alg.

**Figure 13:**
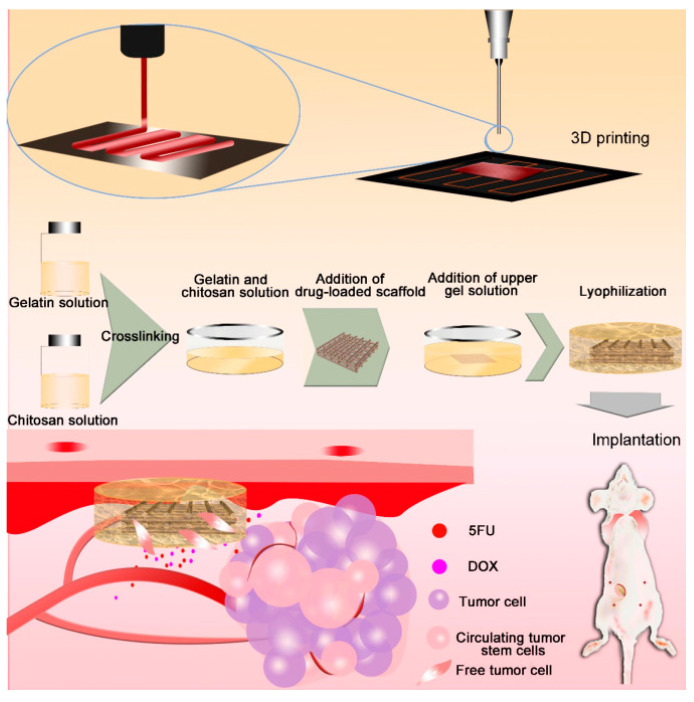
PLGA drug-loaded scaffolds were printed by electrohydrodynamic jet 3D printing and sandwiched in a gelatin-chitosan gel. Scaffolds were implanted in vivo to absorb hemorrhage and cell residues after surgery, and to inhibit cancer cells and circulating tumor cells. Reprinted from [162], Theranostics 2020.

Chitosan and gelatin have also found applications as drug-loaded coatings for 3D printed structures in the design of multifunctional drug delivery devices. For breast cancer treatment, 3D printed scaffolds for sustained anti-cancer drug release and wound healing in postoperative treatment were developed [162]. A PLGA scaffold printed with an electrohydrodynamic jet 3D printing system was loaded with common anti-cancer drugs (5-FU and doxorubicin hydrochloride), and further sandwiched in a Gel-CS hydrogel crosslinked with glutaraldehyde (Figure 13). CS and Gel improved the wound healing process. Additionally, due to the pH-responsiveness of CS, selective sustained release of both 5-FU and doxorubicin in the acidic tumor environment, was achieved in vitro and in vivo. Similarly, Amin Yaravi et al. reported very interesting work involving the coating of a 3D printed titanium disc with up to 20 alternating CS/vancomycin hydrochloride and Gel/BMP-2 layers, through a layer-by-layer method [163]. Depending on the pattern and the number of layers, different release profiles were observed; overall a sustained release over 14 days was achieved, with a small burst release on the first day. It was further demonstrated that the antibacterial effect of vancomycin was observable for up to 14 days. The combination of additive manufacturing with layer-by-layer coating is a promising strategy for the development of multifunctional biomaterials.

Sommer et al. have developed an interesting emulsion-based ink for 3D-printing: an oil-in-water emulsion, stabilized by chitosan-modified silica nanoparticles [164]. Combining an aqueous suspension of CS-modified silica nanoparticles with an oil phase, afforded stable printable emulsions, due to the bridging network of droplets and the gelled silica nanoparticles. The emulsions could be loaded with either hydrophobic or hydrophilic drugs, but also co-loaded with both simultaneously. The authors used a dye to simulate drug encapsulation and precise incorporation of the dye in specific parts of the printed structure and controlled in vitro release was proven, demonstrating the potential application of such formulations in complex drug delivery systems.

## 5. Alginate

Alginate (Alg) is an anionic linear polysaccharide constituted of units of β-d-mannuronic acid (M blocks) and its epimer α-l-guluronic acid (G blocks). Along alginate chains, M blocks and G blocks are separated by MG regions. It is accepted that while G blocks are linked to the gel-forming properties of Alg, MG and M blocks increase the flexibility of the gel [165]. Alg chains can be easily crosslinked in the presence of divalent ions, notably Ca^2+^. Like cellulose and CS, Alg gels are appropriate for 3D printing, a post-printing crosslinking step being often applied to stabilize the printed object [166,167,168]. Several examples where alginate has been combined with cellulose and CS to improve the properties of the gels have already been mentioned. In this section, some complementary interesting reports will be further commented. Due to the good gelling properties of Alg, extrusion-based printing dominates.

Shen et al. encapsulated bacteriophage nanoparticles in an Alg solution to print scaffolds for wound dressings [169]. The phages retained 85–90% of their lytic activity owing to the mild conditions of printing, and an antibacterial activity for 24 h was observed due to their slow release. Human elastin-like polypeptides conjugated to epidermal growth factor (EGF) were adsorbed on 3D printed alginate scaffolds, to prepare patches for the treatment of hard-to-heal skin lesions [170]. In the presence of proteolytic enzymes, EGF is cleaved from the polypeptide and is progressively released from the scaffold. A constant and stable release over 3 weeks from an Alg/sodium hydroxyapatite hydrogel was observed for calcitonin-gene related peptide and naringin [171], while similar results were reported for the release of berberine from an Alg/calcium phosphate scaffold [172].

Pregelatinized starch was incorporated to sodium alginate hydrogels in order to improve the elasticity and adhesive properties of the Alg ink [173]. Patches for topical delivery were further printed. The presence of pregelatinized starch resulted in a reorganization of the structure of alginate causing an increased porosity of the patch and an increased release of Rhodamine B, used as a model in this study. Alg/Gel hydrogels have also been reported [174]. The thermosensitive properties of Gel were exploited and the ink was heated at 37 °C for printing and deposited on a platform cooled at 5 °C. The addition of carrageenan (up to 1.5%) has also been reported to improve the mechanical properties of Alg hydrogels, affording inks with excellent printability and biocompatibility [175]. PEG is another polymer that has been used to control the viscosity of Alg inks [176]. Extracts of Satureja cuneifolia were further incorporated in Alg/PEG inks (9 wt% sodium alginate and 3 wt% PEG) to confer antibacterial properties.

A shape memory hydrogel based on acrylate-modified pluronic F-127 and sodium alginate was studied for the delivery of methotrexate [177]. After printing, the hydrogel was crosslinked by UV irradiation, given a temporary shape, stabilized in that shape by CaCl_2_ crosslinking and finally returned to its initial shape by immersion in Na_2_CO_3_ to remove Ca^2+^ ions. When immersed in distilled water, the temporary shape was recovered. When methotrexate was loaded in the hydrogel, a fast release, appropriate for the release of local anesthesia or the administration of a hemostasis drug, was observed. Finally, it was evidenced that the permanent and temporary shaped scaffolds released the drug at different rates allowing for an additional control point.

As discussed with CS, to control the release of an API, the active ingredient is often encapsulated in polymeric nanoparticles, before being introduced in the printing ink. For example, Song et al. incorporated cyclosporin A-loaded PLGA microspheres in an Alg hydrogel and achieved a sustained release over 4 weeks [178], while Gao et al. observed a sustained release over a month when atorvastatin-loaded PLGA nanoparticles were inserted in a hybrid bioink containing 2% *w*/*w* Alg and 3% *w*/*w* vascular-tissue derived decellularized extracellular matrix [179]. In the same vein, Liu et al. developed an alginate/collagen hydrogel containing HAp nanoparticles and enrofloxacin-loaded PCL microspheres, for the printing of a long-term antimicrobial scaffold [180]. Alg and Col were crosslinked by calcium ions and genipin, respectively, forming a double interpenetrating network. A sustained released of enrofloxacin was observed, and, more important, a long-term antibacterial activity against *E. coli* and *S. aureus*, demonstrating a promising potential for further applications.

Interesting—in terms of design and results—drug delivery devices with more complex architectures for sequential or on-demand API delivery have also been reported. For example, Akkineni et al. have described a biphasic scaffold composed of a calcium phosphate cement and a vascular endothelial growth factor (VEGF)-releasing Alg-gellan gum hydrogel [181]. The scaffold, printed by employing a multi-channel dosing unit, was designed with an increasing number of VEGF-loaded hydrogel strands going from the outer surface to the center of the scaffold in order to delay its release. Overall, a sustained release of VEGF over a week was observed and it was demonstrated that the activity of the released VEGF was in the same range with freshly added VEGF.

Core-shell fibers with different outer shells and inner cores, which can be easily obtained by coaxial printing, is another design that has afforded very promising results. Indeed, this design allows for simultaneous multiple drug loading, spatially controlled localization of the drugs within the delivery system and controlled, even sequential, drug release. For example, one could expect a drug loaded in the outer shell to be released before one located in the core. Additionally, very different polymeric materials, e.g., hydrophilic/hydrophobic or thermoplastics/hydrogels can be readily combined.

**Figure 14:**
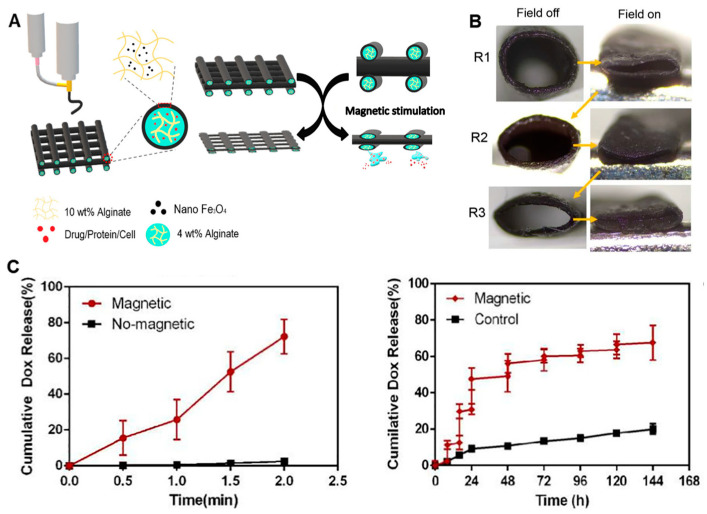
(**A**) Scheme of 3D printing magnetically driven drug delivery system. Under magnetic stimulation the drug-loaded core was extruded from the open ends of the scaffolds achieving drug release. (**B**) The images of the hollow alginate/iron oxide nanoparticle scaffold under magnetic field off/on for three cycles. (**C**) Cumulative doxorubicin release from the scaffold under the magnetic stimulation. Left: for 2 min. Right: over 144 h and under repeated magnetic field on/off. Reprinted with permission from [187], Copyright 2020, Elsevier.

Falcone et al. used coaxial printing with a sacrificial core to prepare hollow floating tubes. Alg was in the outer shell and was in situ crosslinked and drug-loaded by the inner core, that was composed of the crosslinking agent (CaCl_2_) and the drug (propanolol hydrochloride) [182]. PCL hollow shells loaded with bevacizumab were obtain by coaxial printing with a sacrificial core [183]. The sacrificial core was rinsed and replaced by an Alg gel loaded with dexamethasone. Overall dual-drugloaded injectable rods were obtained and time-controlled release of the two drugs was achieved. Do et al. have reported a delivery device composed of a 3D-printed outer Alg tube and an inner PLGA core for the sequential delivery of APIs, that could be potentially used as a drug eluting implant in cancer therapy [184]. The proof of concept was demonstrated with two fluorophores: fluorescein and rhodamine. The release studies demonstrated a burst release of the fluorophore contained in the outer Alg tube over 24 h, and a controlled release of the drug contained in the PLGA core starting on the second day and extending for another 3–4 days. In a similar concept, Akkineni et al. reported the coaxial printing of a sodium alginate paste prepared with PVA, yielding a stiff shell, combined with various softer hydrogels as the core material [185]. Both the shell and the core were loaded with protein drugs (BSA, VEGF, BMP) and dual release was achieved. Wei et al. developed a 3D-printed core-shell implant for the near infrared (NIR)-triggered release of doxorubicin, combining photothermal therapy with chemotherapy [186]. Core-shell fibers were prepared by the coaxial printing of a Gel/doxorubicin core and a polydopamine/Alg shell. The concentration of the Gel core was 70% wt., thus maintaining a gel state at body temperature. When the fibers were irradiated with NIR light, an important temperature increase was caused due to the photothermal effect of polydopamine. As a result, gelatin transformed to sol leaking out of the open ends of the fibers thus releasing doxorubicin. Tumor growth was effectively inhibited in in vivo studies. Similarly, Wang et al. elaborated a magnetically controlled, on-demand drug-delivery platform based on 3D-printed fibers, illustrated in Figure 14 [187]. Alginate core-shell fibers were prepared by co-axial 3D printing, by combining an alginate/iron oxide nanoparticle (ION) hydrogel for the outer shell and a drug-loaded sodium alginate hydrogel for the fiber core (doxorubicin and bovine serum albumin were studied). Upon exposure to magnetic field, the fibers were deformed releasing the inner drug-loaded hydrogel. The composition of the outer Alg/iron oxide nanoparticle hydrogel and the crosslinking conditions were optimized in order to obtain structures with satisfying structural stability, but soft enough to be repeatedly and reversibly deformed under a magnetic field. For the optimal conditions (10 wt.% Alg and 13% ION; crosslinked by 0.1 M CaCl_2_ for 10 s), a linear deformation with time was observed upon magnetic exposure (for the first 2 min), resulting in a linear drug release.

## 6. Conclusions

The aim of the present review is to highlight the recent progresses in the printing of polysaccharides for drug delivery applications. Three-dimensional printing is a powerful method for personalized medical treatment. Three-dimensional printing can not only satisfy personalization requirements in terms of shape/form, e.g., for wound dressings, but most importantly, drug dosing and drug release can be readily tailored according to the specific needs of a patient. For example, drug release can be simply modified by changing the infill percentage or geometry during printing. The ultimate goal of any drug delivery system is to precisely control drug release, either immediate or progressive over a certain period of time. With 3D printing, APIs can be loaded in specific parts of the printed structure, allowing for a programmable release. Moreover, more complex architectures can be easily and reproducibly obtained by 3D printing, allowing for a better control and tailoring of drug release.

Polysaccharides are natural polymers, ubiquitous, versatile and rather inexpensive, which can be adapted to different printing techniques as needed. Due to their biocompatibility and biodegradability, they are particularly appropriate for drug delivery applications. Their intrinsic properties can be easily tuned through chemical modification (increase solubility, change hydrophilic/hydrophobic balance, etc.) and further improved by combining them with other, carefully chosen, natural or synthetic polymers. Polysaccharides are susceptible to crosslinking, that can be implemented to maintain the structural integrity of the printed objects, increase their mechanical properties or delay their degradation. Moreover, polysaccharides bearing ionic groups can exhibit a responsive behavior, e.g., pH-responsive for chitosan, that allows for an additional level of control [188]. To conclude, polysaccharides and 3D printing have a promising future ahead of them, both for the large-scale production of drugs formulations or for the smaller-scale preparation of patient-tailored medications.

## Figures and Tables

**Table 1:** Common polysaccharides used in 3D printing.

	Charge	Functionalities
**Cellulose**	neutral	–OH
	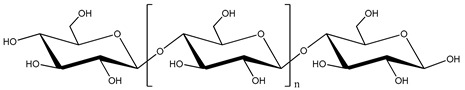	
**Chitosan**	positive	–NH_3_^+^, –OH
	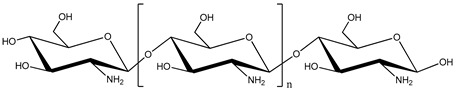	
**Sodium alginate**	negative	–OH, COO^−^
	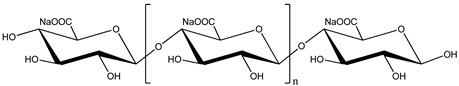	
**Hyaluronic acid**	negative	–OH, COO^−^
	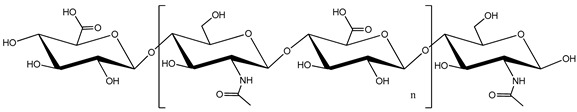	
**Pectin**	negative	–OH, COO^−^, COOCH_3_
	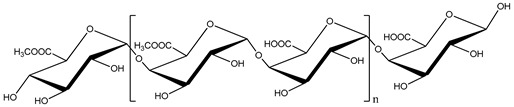	

## Abbreviations

5-FU	5-fluorouracil
ALG	Alginate
BC	Bacterial cellulose
BMP	Bone morphogenic protein
BSA	Bovine serum albumin
CAD	Computer aided design
CMC	Carboxymethyl cellulose
CS	Chitosan
EC	Ethyl cellulose
EGF	Epidermal growth factor
FDA	Food and Drug Administration
FDM	Fused deposition modeling
FBS	Fetal bovine serum
Gel	Gelatin
GRAS	Generally regarded as safe
HAc	Hyaluronic acid
HAp	Hydroxyapatite
HPMC	Hydroxypropyl methylcellulose
HME	Hot-melt extrusion
HPC	Hydroxypropyl cellulose
HPMCAS	Hydroxypropyl methyl cellulose acetate succinate
ION	Iron oxide nanoparticle
LDM	Liquid deposition modeling
nanoHAp	Nanohydroxyapatite
NIR	Near infrared
PCL	Poly(caprolactone)
Pec	Pectin
PgGA	Poly(gamma-glutamic acid)
PLA	Poly(lactic acid)
rhBMP	Recombinant human bone morphogenic proteins
SEM	Scanning electron microscopy
SSE	Semi-solid extrusion
SLS	Selective laser sintering
TEMPO	2,2,6,6-tetramethylpiperidinyl-1-oxyl
TOBC	TEMPO-oxidized BC
TIJ	Thermal inkjet
